# (*E*)-4-Amino-*N*′-(5-chloro-2-hy­droxy­benzyl­idene)benzohydrazide

**DOI:** 10.1107/S1600536812025974

**Published:** 2012-06-16

**Authors:** Hadi Kargar, Reza Kia, Muhammad Nawaz Tahir

**Affiliations:** a Department of Chemistry, Payame Noor University, PO BOX 19395-3697 Tehran, I. R. of IRAN; b Department of Chemistry, Science and Research Branch, Islamic Azad University, Tehran, Iran; c Department of Physics, University of Sargodha, Punjab, Pakistan

## Abstract

In the title hydrazide Schiff base compound, C_14_H_12_ClN_3_O_2_, the conformation around the C=N double bond is *E*. The dihedral angle between the benzene rings is 41.57 (14) Å. An intra­molecular O—H⋯N hydrogen bond makes an *S*(6) ring motif. In the crystal, mol­ecules are linked by N—H⋯O (bifurcated acceptor) and N—H⋯N hydrogen bonds, forming chains along the *a* axis. The inter­esting feature of the crystal structure is the short inter­molecular C⋯O [3.216 (3), 3.170 (3), and 2.992 (3) Å] contacts, one of which is significantly shorter than the sum of the van der Waals radii of these atoms [3.22 Å].

## Related literature

For the coordination chemistry of Schiff base and hydrazone derivatives, see: Kucukguzel *et al.* (2006 ▶); Karthikeyan *et al.* (2006 ▶). For 4-amino­benzohydrazide-derived Schiff base structures, see: Xu (2012 ▶); Shi & Li (2012 ▶); Bakir & Green (2002 ▶). For standard bond lengths, see: Allen *et al.* (1987 ▶). For hydrogen-bond motifs, see: Bernstein *et al.* (1995 ▶). For van der Waals radii, see: Bondi (1964 ▶).
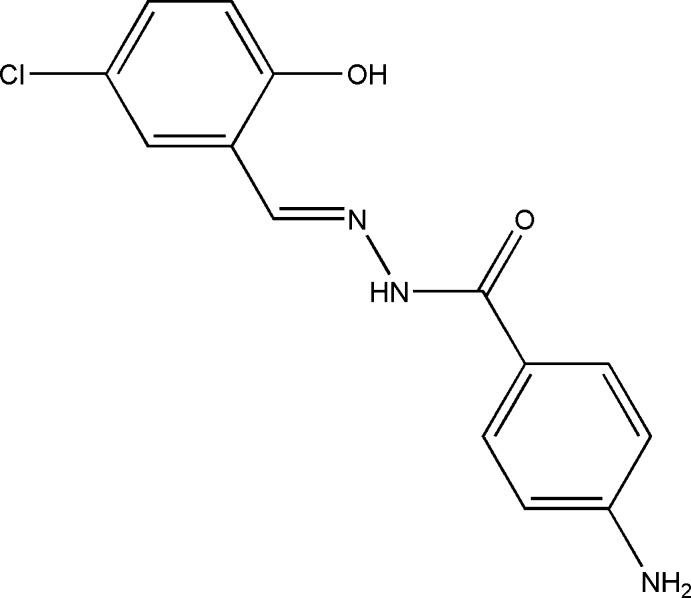


## Experimental

### 

#### Crystal data

C_14_H_12_ClN_3_O_2_
*M* *_r_* = 289.72
Orthorhombic, 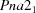

*a* = 9.4243 (8) Å
*b* = 9.7975 (9) Å
*c* = 14.1924 (10) Å
*V* = 1310.45 (19) Å^3^
*Z* = 4
Mo *K*α radiation
μ = 0.30 mm^−1^
*T* = 296 K
0.30 × 0.25 × 0.22 mm

#### Data collection

Bruker SMART APEXII CCD area-detector diffractometer
Absorption correction: multi-scan (*SADABS*; Bruker, 2005 ▶) *T*_min_ = 0.916, *T*_max_ = 0.938
5390 measured reflections
2157 independent reflections
1871 reflections with *I* > 2σ(*I*)
*R*_int_ = 0.023

#### Refinement

*R*[*F* ^2^ > 2σ(*F* ^2^)] = 0.035
*wR*(*F* ^2^) = 0.084
*S* = 1.04
2157 reflections
182 parameters
1 restraint
H-atom parameters constrained
Δρ_max_ = 0.25 e Å^−3^
Δρ_min_ = −0.28 e Å^−3^
Absolute structure: Flack (1983 ▶), 1661 Friedel pairs
Flack parameter: −0.02 (8)

### 

Data collection: *APEX2* (Bruker, 2005 ▶); cell refinement: *SAINT* (Bruker, 2005 ▶); data reduction: *SAINT*; program(s) used to solve structure: *SHELXS97* (Sheldrick, 2008 ▶); program(s) used to refine structure: *SHELXL97* (Sheldrick, 2008 ▶); molecular graphics: *SHELXTL* (Sheldrick, 2008 ▶)’; software used to prepare material for publication: *SHELXTL* and *PLATON* (Spek, 2009 ▶).

## Supplementary Material

Crystal structure: contains datablock(s) global, I. DOI: 10.1107/S1600536812025974/su2449sup1.cif

Structure factors: contains datablock(s) I. DOI: 10.1107/S1600536812025974/su2449Isup2.hkl

Supplementary material file. DOI: 10.1107/S1600536812025974/su2449Isup3.cml

Additional supplementary materials:  crystallographic information; 3D view; checkCIF report

## Figures and Tables

**Table 1:** Hydrogen-bond geometry (Å, °)

*D*—H⋯*A*	*D*—H	H⋯*A*	*D*⋯*A*	*D*—H⋯*A*
O1—H1⋯N1	0.82	1.87	2.584 (3)	145
N2—H2⋯O2^i^	0.98	1.98	2.951 (3)	169
N3—H3*B*⋯O2^ii^	0.96	2.09	3.004 (3)	159

Symmetry codes: (i) 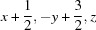; (ii) 
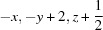
.

**Fig. 1.**
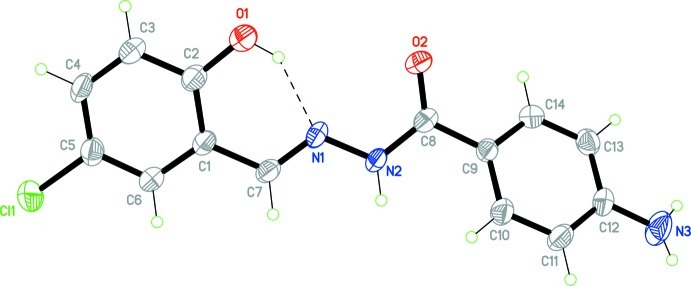
A view of the molecular structure of the title compound, showing 40% probability displacement ellipsoids and the atom numbering. The dashed lines show the intramolecular O-H···N hydrogen bonds (see Table 1 for details).

**Fig. 2.**
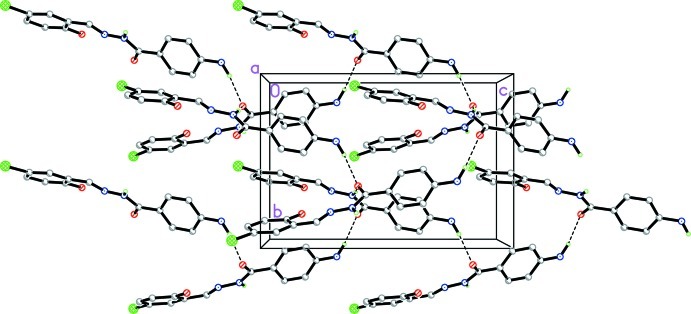
A view along the a axis of crystal packing of the title compound, showing how the molecules are linked via N—H···O hydrogen bonds (dashed lines; see Table 1 for details). Only the H atoms involved in these interactions are shown.

## supplementary crystallographic information

### Comment

Schiff bases are one of the most prevalent mixed-donor ligands in the field of coordination chemistry. They play an important role in the development of coordination chemistry related to catalysis and magnetism, and supramolecular architectures (Karthikeyan *et al.*, 2006; Kucukguzel *et al.*, 2006). Structures of Schiff bases derived from substituted 4-aminobenzohydrazide have been reported earlier (Xu, 2012; Shi & Li, 2012; Bakir & Green, 2002). In order to explore the structure of new Schiff base derivatives, the title compound was prepared and characterized crystallographically.

The title molecule, Fig. 1, has an *E* conformation around C═N double bond. The bond lengths (Allen *et al.*, 1987) and angles are within normal ranges and are comparable to those reported for related structures (Xu, 2012; Shi & Li, 2012; Bakir & Green, 2002). The dihedral angle between the substituted phenyl rings is 41.57 (14)Å. An intramolecular O—H···N hydrogen bond makes an *S(6)* ring motif (Bernstein *et al.*, 1995).

In the crystal, molecules are linked through N—H···O (bifurcated acceptor) and N—H···N hydrogen bonds forming one-dimensional chains along the *a* axis. An interesting feature of the crystal structure is the short intermolecular C1···O1^iii^ [3.216 (3) Å; (iii) -1/2 + *x*, 3/2 - *y*, *z*], C6···O1^iii^ [3.170 (3) Å], and C7···O1^iii^ [2.992 (3) Å] contacts, in which one of them is significantly shorter than the sum of the van der Waals radii of these atoms [3.22Å; Bondi, 1964].

### Experimental

The title compound was synthesized by adding 1 mmol of methyl 4-aminobenzoate to a solution of 5-chlorosalicylaldehyde (1 mmol) in methanol (30 ml). The mixture was refluxed with stirring for 30 min and after cooling to room temperature a light-yellow precipitate was filtered and washed with diethylether and dried in air. Light-yellow prismatic crystals of the title compound, suitable for *X*-ray structure analysis, were recrystallized from ethanol by slow evaporation of the solvents at room temperature over several days.

### Refinement

The N-bound H atoms were located in a difference Fourier map and were constrained to ride on their parent atoms with U_iso_(H) = 1.2U_eq_(N). The OH H atom was positioned by a freely rotating OH group model: O-H = 0.82 Å with U_iso_(H) = 1.5U_eq_(O). The C-bound H atoms were included in calculated positions and treated as riding atoms: C—H = 0.93 Å with U_iso_(H) = 1.2U_eq_(C).

### Crystal data

**Table:**

C_14_H_12_ClN_3_O_2_	*F*(000) = 600
*M**_r_* = 289.72	*D*_x_ = 1.468 Mg m^−^^3^
Orthorhombic, *P**n**a*2_1_	Mo *K*α radiation, λ = 0.71073 Å
Hall symbol: P 2c -2n	Cell parameters from 1125 reflections
*a* = 9.4243 (8) Å	θ = 2.5–27.4°
*b* = 9.7975 (9) Å	µ = 0.30 mm^−^^1^
*c* = 14.1924 (10) Å	*T* = 296 K
*V* = 1310.45 (19) Å^3^	Prism, light-yellow
*Z* = 4	0.30 × 0.25 × 0.22 mm

### Data collection

**Table:**

Bruker SMART APEXII CCD area-detector diffractometer	2157 independent reflections
Radiation source: fine-focus sealed tube	1871 reflections with *I* > 2σ(*I*)
Graphite monochromator	*R*_int_ = 0.023
φ and ω scans	θ_max_ = 27.2°, θ_min_ = 2.5°
Absorption correction: multi-scan (*SADABS*; Bruker, 2005)	*h* = −8→12
*T*_min_ = 0.916, *T*_max_ = 0.938	*k* = −12→9
5390 measured reflections	*l* = −14→17

### Refinement

**Table:**

Refinement on *F*^2^	Secondary atom site location: difference Fourier map
Least-squares matrix: full	Hydrogen site location: inferred from neighbouring sites
*R*[*F*^2^ > 2σ(*F*^2^)] = 0.035	H-atom parameters constrained
*wR*(*F*^2^) = 0.084	*w* = 1/[σ^2^(*F*_o_^2^) + (0.0457*P*)^2^ + 0.1075*P*] where *P* = (*F*_o_^2^ + 2*F*_c_^2^)/3
*S* = 1.04	(Δ/σ)_max_ < 0.001
2157 reflections	Δρ_max_ = 0.25 e Å^−^^3^
182 parameters	Δρ_min_ = −0.28 e Å^−^^3^
1 restraint	Absolute structure: Flack (1983), 1661 Friedel pairs
Primary atom site location: structure-invariant direct methods	Flack parameter: −0.02 (8)

### Special details

**Table:**

Geometry. All esds (except the esd in the dihedral angle between two l.s. planes) are estimated using the full covariance matrix. The cell esds are taken into account individually in the estimation of esds in distances, angles and torsion angles; correlations between esds in cell parameters are only used when they are defined by crystal symmetry. An approximate (isotropic) treatment of cell esds is used for estimating esds involving l.s. planes.
Refinement. Refinement of F^2^ against ALL reflections. The weighted R-factor wR and goodness of fit S are based on F^2^, conventional R-factors R are based on F, with F set to zero for negative F^2^. The threshold expression of F^2^ > 2sigma(F^2^) is used only for calculating R-factors(gt) etc. and is not relevant to the choice of reflections for refinement. R-factors based on F^2^ are statistically about twice as large as those based on F, and R- factors based on ALL data will be even larger.

### Fractional atomic coordinates and isotropic or equivalent isotropic displacement parameters (Å^2^)

**Table:**

	*x*	*y*	*z*	*U*_iso_*/*U*_eq_	
Cl1	0.25011 (8)	0.53141 (8)	−0.12609 (6)	0.0608 (2)	
O1	−0.18920 (19)	0.6731 (2)	0.14787 (13)	0.0576 (6)	
H1	−0.1557	0.6890	0.2001	0.086*	
O2	−0.14326 (17)	0.8456 (2)	0.38803 (12)	0.0433 (5)	
N1	0.0101 (2)	0.6912 (2)	0.27231 (13)	0.0357 (5)	
N2	0.0530 (2)	0.7181 (2)	0.36316 (14)	0.0348 (5)	
H2	0.1501	0.6912	0.3791	0.042*	
N3	0.1425 (3)	0.8793 (3)	0.79456 (16)	0.0608 (7)	
H3A	0.1989	0.8266	0.8306	0.073*	
H3B	0.1185	0.9611	0.8284	0.073*	
C1	0.0540 (3)	0.6151 (3)	0.11808 (16)	0.0343 (6)	
C2	−0.0834 (3)	0.6413 (3)	0.08747 (18)	0.0401 (6)	
C3	−0.1161 (3)	0.6341 (3)	−0.00784 (18)	0.0452 (7)	
H3	−0.2084	0.6516	−0.0277	0.054*	
C4	−0.0146 (3)	0.6016 (3)	−0.07299 (17)	0.0436 (7)	
H4	−0.0368	0.5995	−0.1368	0.052*	
C5	0.1210 (3)	0.5721 (3)	−0.04292 (18)	0.0409 (6)	
C6	0.1556 (3)	0.5765 (3)	0.05102 (18)	0.0413 (6)	
H6	0.2469	0.5538	0.0703	0.050*	
C7	0.0965 (3)	0.6349 (3)	0.21540 (17)	0.0371 (6)	
H7	0.1856	0.6069	0.2359	0.045*	
C8	−0.0326 (3)	0.7949 (3)	0.41874 (17)	0.0337 (6)	
C9	0.0153 (2)	0.8152 (3)	0.51641 (17)	0.0327 (6)	
C10	0.1079 (3)	0.7271 (3)	0.56124 (18)	0.0451 (7)	
H10	0.1439	0.6526	0.5284	0.054*	
C11	0.1479 (3)	0.7471 (3)	0.65323 (18)	0.0492 (7)	
H11	0.2087	0.6851	0.6820	0.059*	
C12	0.0986 (3)	0.8585 (3)	0.70364 (17)	0.0397 (6)	
C13	0.0065 (3)	0.9481 (3)	0.65961 (18)	0.0422 (6)	
H13	−0.0274	1.0237	0.6922	0.051*	
C14	−0.0354 (3)	0.9260 (3)	0.56763 (19)	0.0408 (6)	
H14	−0.0987	0.9863	0.5394	0.049*	

### Atomic displacement parameters (Å^2^)

**Table:**

	*U*^11^	*U*^22^	*U*^33^	*U*^12^	*U*^13^	*U*^23^
Cl1	0.0686 (4)	0.0770 (5)	0.0367 (3)	0.0182 (4)	0.0054 (4)	−0.0052 (4)
O1	0.0410 (10)	0.0937 (18)	0.0380 (11)	0.0132 (11)	−0.0035 (9)	−0.0045 (12)
O2	0.0346 (9)	0.0579 (12)	0.0375 (11)	0.0050 (8)	−0.0085 (8)	0.0032 (10)
N1	0.0394 (11)	0.0443 (13)	0.0233 (10)	−0.0043 (9)	−0.0059 (9)	0.0007 (9)
N2	0.0343 (10)	0.0477 (13)	0.0223 (10)	0.0009 (10)	−0.0055 (9)	0.0001 (10)
N3	0.0859 (18)	0.0627 (19)	0.0338 (13)	0.0048 (14)	−0.0158 (12)	−0.0128 (13)
C1	0.0382 (14)	0.0355 (14)	0.0291 (13)	−0.0005 (11)	−0.0069 (11)	0.0009 (12)
C2	0.0394 (14)	0.0466 (18)	0.0342 (13)	−0.0009 (13)	−0.0055 (12)	0.0016 (13)
C3	0.0443 (16)	0.0535 (18)	0.0377 (15)	0.0016 (13)	−0.0122 (13)	−0.0011 (13)
C4	0.0592 (18)	0.0452 (16)	0.0265 (13)	0.0014 (13)	−0.0117 (12)	−0.0025 (13)
C5	0.0526 (16)	0.0383 (16)	0.0317 (13)	0.0027 (12)	−0.0010 (12)	−0.0010 (13)
C6	0.0414 (15)	0.0480 (16)	0.0345 (14)	0.0057 (13)	−0.0091 (11)	−0.0008 (13)
C7	0.0324 (13)	0.0469 (17)	0.0320 (13)	0.0001 (12)	−0.0075 (11)	0.0028 (13)
C8	0.0327 (13)	0.0370 (14)	0.0313 (13)	−0.0053 (11)	−0.0050 (11)	0.0045 (12)
C9	0.0303 (13)	0.0398 (14)	0.0279 (12)	−0.0013 (11)	−0.0008 (10)	0.0000 (11)
C10	0.0593 (17)	0.0438 (17)	0.0323 (15)	0.0127 (14)	−0.0052 (13)	−0.0053 (13)
C11	0.0669 (18)	0.0450 (16)	0.0357 (14)	0.0155 (14)	−0.0133 (14)	0.0005 (13)
C12	0.0451 (15)	0.0457 (17)	0.0283 (12)	−0.0045 (13)	0.0010 (12)	−0.0025 (13)
C13	0.0424 (14)	0.0446 (15)	0.0395 (15)	0.0015 (13)	0.0030 (12)	−0.0095 (14)
C14	0.0367 (13)	0.0452 (16)	0.0406 (15)	0.0060 (13)	−0.0019 (12)	0.0021 (13)

### Geometric parameters (Å, º)

**Table:**

Cl1—C5	1.742 (3)	C4—C5	1.378 (4)
O1—C2	1.351 (3)	C4—H4	0.9300
O1—H1	0.8200	C5—C6	1.373 (3)
O2—C8	1.235 (3)	C6—H6	0.9300
N1—C7	1.272 (3)	C7—H7	0.9300
N1—N2	1.377 (3)	C8—C9	1.471 (3)
N2—C8	1.357 (3)	C9—C10	1.382 (4)
N2—H2	0.9783	C9—C14	1.392 (4)
N3—C12	1.370 (3)	C10—C11	1.373 (4)
N3—H3A	0.9001	C10—H10	0.9300
N3—H3B	0.9616	C11—C12	1.385 (4)
C1—C2	1.390 (3)	C11—H11	0.9300
C1—C6	1.402 (3)	C12—C13	1.383 (4)
C1—C7	1.451 (3)	C13—C14	1.381 (4)
C2—C3	1.389 (4)	C13—H13	0.9300
C3—C4	1.368 (4)	C14—H14	0.9300
C3—H3	0.9300		
			
C2—O1—H1	109.5	C1—C6—H6	119.8
C7—N1—N2	119.3 (2)	N1—C7—C1	119.1 (2)
C8—N2—N1	118.43 (19)	N1—C7—H7	120.5
C8—N2—H2	124.8	C1—C7—H7	120.5
N1—N2—H2	116.1	O2—C8—N2	121.4 (2)
C12—N3—H3A	128.9	O2—C8—C9	122.5 (2)
C12—N3—H3B	121.5	N2—C8—C9	116.1 (2)
H3A—N3—H3B	109.5	C10—C9—C14	117.6 (2)
C2—C1—C6	118.3 (2)	C10—C9—C8	122.9 (2)
C2—C1—C7	122.0 (2)	C14—C9—C8	119.5 (2)
C6—C1—C7	119.6 (2)	C11—C10—C9	121.5 (3)
O1—C2—C3	117.8 (2)	C11—C10—H10	119.3
O1—C2—C1	122.1 (2)	C9—C10—H10	119.3
C3—C2—C1	120.1 (2)	C10—C11—C12	120.8 (3)
C4—C3—C2	121.0 (2)	C10—C11—H11	119.6
C4—C3—H3	119.5	C12—C11—H11	119.6
C2—C3—H3	119.5	N3—C12—C13	121.4 (3)
C3—C4—C5	119.2 (2)	N3—C12—C11	120.2 (3)
C3—C4—H4	120.4	C13—C12—C11	118.5 (2)
C5—C4—H4	120.4	C14—C13—C12	120.5 (3)
C6—C5—C4	121.0 (2)	C14—C13—H13	119.8
C6—C5—Cl1	119.9 (2)	C12—C13—H13	119.8
C4—C5—Cl1	119.1 (2)	C13—C14—C9	121.2 (2)
C5—C6—C1	120.4 (2)	C13—C14—H14	119.4
C5—C6—H6	119.8	C9—C14—H14	119.4
			
C7—N1—N2—C8	170.8 (2)	N1—N2—C8—O2	−3.4 (3)
C6—C1—C2—O1	177.0 (3)	N1—N2—C8—C9	177.3 (2)
C7—C1—C2—O1	−7.1 (4)	O2—C8—C9—C10	157.6 (3)
C6—C1—C2—C3	−2.4 (4)	N2—C8—C9—C10	−23.1 (3)
C7—C1—C2—C3	173.6 (3)	O2—C8—C9—C14	−21.3 (4)
O1—C2—C3—C4	−179.6 (3)	N2—C8—C9—C14	158.0 (2)
C1—C2—C3—C4	−0.2 (5)	C14—C9—C10—C11	0.5 (4)
C2—C3—C4—C5	1.8 (4)	C8—C9—C10—C11	−178.4 (3)
C3—C4—C5—C6	−0.8 (4)	C9—C10—C11—C12	−1.4 (5)
C3—C4—C5—Cl1	−179.8 (2)	C10—C11—C12—N3	−178.2 (3)
C4—C5—C6—C1	−1.8 (4)	C10—C11—C12—C13	1.0 (4)
Cl1—C5—C6—C1	177.2 (2)	N3—C12—C13—C14	179.4 (3)
C2—C1—C6—C5	3.4 (4)	C11—C12—C13—C14	0.3 (4)
C7—C1—C6—C5	−172.7 (3)	C12—C13—C14—C9	−1.1 (4)
N2—N1—C7—C1	−175.9 (2)	C10—C9—C14—C13	0.7 (4)
C2—C1—C7—N1	−7.4 (4)	C8—C9—C14—C13	179.7 (2)
C6—C1—C7—N1	168.5 (2)		

### Hydrogen-bond geometry (Å, º)

**Table:**

*D*—H···*A*	*D*—H	H···*A*	*D*···*A*	*D*—H···*A*
O1—H1···N1	0.82	1.87	2.584 (3)	145
N2—H2···O2^i^	0.98	1.98	2.951 (3)	169
N3—H3*B*···O2^ii^	0.96	2.09	3.004 (3)	159

Symmetry codes: (i) *x*+1/2, −*y*+3/2, *z*; (ii) −*x*, −*y*+2, *z*+1/2.
